# Survival of Unstressed and Acid-, Cold-, and Starvation-Stress-Adapted *Listeria monocytogenes* in Ham Extract with Hops Beta Acids and Consumer Acceptability of HBA on Ready-to-Eat Ham

**DOI:** 10.1155/2015/817042

**Published:** 2015-10-11

**Authors:** Li Wang, Cangliang Shen

**Affiliations:** ^1^State Key Laboratory of Food Science and Technology, National Engineering Laboratory for Cereal Fermentation Technology, School of Food Science and Technology, Jiangnan University, Wuxi, Jiangsu 214122, China; ^2^Division of Animal and Nutritional Sciences, West Virginia University, Morgantown, WV 26506, USA

## Abstract

The efficacy of hops beta acids (HBA) against unstressed and stress-adapted *Listeria monocytogenes* in ham extract and the consumers' acceptability of HBA on ready-to-eat (RTE) hams were investigated. Unstressed or acid-, cold-, or starvation-stress-adapted *L. monocytogenes* was inoculated (1.3–1.5 log CFU/mL) into 10% ham extract, without (control) or with HBA (4.44 or 10.0 *µ*g/mL). Survival/growth of the pathogen during storage (7.2°C, 26 days) was monitored periodically. Sensory evaluation (30 participants, 9-point hedonic scale) was performed with hams dipped into 0.05, 0.11, and 0.23% HBA solution. Ham extracts without HBA supported rapid growth of unstressed and stress-adapted cells with growth rates of 0.39–0.71 log CFU/mL/day and lag phases of 0–3.26 days. HBA inhibited growth of unstressed *L. monocytogenes* by slowing (*P* < 0.05) growth rate (0.24–0.29 log CFU/mL/day) and increasing (*P* < 0.05) length of the lag phase (3.49–12.98 days) compared to control. Acid-, cold-, or starvation-stress-adapted cells showed cross protection against HBA with greater (*P* < 0.05) growth rates (0.44–0.66 log CFU/mL/day) and similar or shorter lag phases (0–5.44 days) than unstressed cells. HBA did not (*P* > 0.05) affect sensory attributes of RTE ham. These results are useful for RTE meat processors to develop operational protocols using HBA to control *L. monocytogenes*.

## 1. Introduction

*Listeria monocytogenes*, a Gram-positive, non-endospore-forming, facultative, and psychrotrophic foodborne pathogen, causes listeriosis, which is an important public health problem in the United States [1]. The groups with high risk to listeriosis include older adults, pregnant women, newborn babies, and immune-compromised patients [2]. The “zero-tolerance” policy was established by the U.S. Department of Agriculture Food Safety and Inspection Service (USDA-FSIS) in early 1990s for guidance and standards of testing and control of* L. monocytogenes* in ready-to-eat (RTE) meat products [3]. However, from 1998 to 2002, several multistate outbreaks of listeriosis associated with RTE deli meat products occurred in the United States [4–7]. Immediately after these outbreaks, in 2003, the USDA-FSIS began to require RTE meat processors to execute “three alternatives” for* L. monocytogenes* control. Alternatives 1 and 2 require the use of postlethality treatments including the application of antimicrobials [8]. However, since then, the detected presence of* L. monocytogenes* in RTE meat and poultry products has decreased gradually. Contamination of* L. monocytogenes* in deli meat still costs approximately $1.1 billion and 4,000 deaths each year in USA [9]. Therefore, processors of RTE products should continue to develop effective approaches for control of* Listeria* during RTE meat processing [9].

Various stresses adapted cells often develop and survive in the meat processing environment or on meat surfaces. For instance, increasing use of acid treatments, such as lactic or acetic acids, on beef or poultry carcasses induced the development of acid-stress-adapted pathogenic cells. Similarly, low temperature food storage may lead to cold-stress-resistant cells; exposure of cells to poor nutrition areas such as facility surfaces, walls, and floors may induce starvation-stressed cells [10]. The efficacy of antimicrobials is sometimes decreased due to the generation of cross protection when a stress-adapted cell is subsequently exposed to a sequentially sublethal stress [11]. Therefore, the stress response of foodborne pathogens in different food systems has recently received much research attention.

Hops beta acids (HBA), extracted from hops flowers, with primary components of lupulone (C_27_H_38_O_4_), colupulone (C_26_H_37_O_4_), and adlupulone (C_27_H_38_O_4_) have been approved by the USDA-FSIS and US-Food and Drug Administration to be used as generally recognized as safe (GRAS) antimicrobial agents on cooked meat surfaces and in casings [12, 13]. Previous studies by Shen and Sofos showed that HBA can efficiently inhibit growth of* L. monocytogenes *in a culture broth medium [14] and on frankfurters under vacuum packaged storage [15]. However, no published literature addressed the antilisterial activity of HBA in food systems, particularly with various stresses adapted* L. monocytogenes*. In addition, hops have been well known for a special dark brownish color and bitter taste that contribute to the beer brewing process, and no published studies evaluated consumer acceptability of application of HBA on RTE meat products.

Therefore, the objectives of this study were to evaluate the efficacy of HBA to inactivate unstressed and acid-, cold-, and starvation-stress-adapted* L. monocytogenes* in ham extract during storage at 7.2°C and the sensory acceptability of HBA applied on commercial RTE hams.

## 2. Materials and Methods

### 2.1. Bacterial Strains and Preparation of Unstressed or Stress-Adapted Cells

The 4-strain mixture of* L. monocytogenes* (kindly provided by Dr. Joshua Gurlter, at USDA-ARS, Wyndmoor, PA) used in this study included ATCC 15213, Scott A 724 (Massachusetts meat outbreak, serotype 4b), L499 (sliced turkey outbreak strain, human isolate, serotype 1/2a), and L502 (chocolate milk outbreak, serotype 1/2b). Each* L. monocytogenes* strain, taken from the −20°C stock culture, was first activated by streak plating onto PALCAM agar (Difco, BD, Sparks, MD) and then incubated at 35°C for 48 h. The procedure of preparing unstressed and three types of stress-adapted cells followed the previous study [16]. To prepare the unstressed cells, a single colony of each* L. monocytogenes* strain was cultured and subcultured (35°C, 24 h) in 10 mL of glucose-free tryptic soy broth with yeast extract (TSB-G + YE). To prepare the acid-stress-adapted cells, the TSB-G + YE cultured (35°C, 24 h) cell suspension was subcultured (0.1 mL, 24 h at 35°C) into 10 mL of TSB-G + YE supplemented with 1% glucose. For preparation of cold- and starvation-stress-adapted cells, the TSB-G + YE cultured (35°C, 24 h) cell suspension was first triplicate-washed in 10 mL phosphate-buffered saline (PBS, pH 7.4), resuspended in 10 mL of TSB-G + YE with storage at 4°C for 7 days, and resuspended in 10 mL of 0.85% NaCl solution with storage at 35°C for 48 hours, respectively. Prior to the experiment, the unstressed or acid-, cold-, or starvation-stress-adapted cells of each strain were washed by centrifuging at 4,629 ×g for 15 min at 4°C three times with 10 mL PBS. The cell pellets were resuspended and serially diluted in PBS to reach a target inoculation level of 1.3 to 1.6 log CFU/mL when 0.1 mL of inoculum was added into ham extract solutions.

### 2.2. Ham Extract Preparation and Inoculation

Fresh uncured ham was purchased from a local supermarket at Wuxi, Jiangsu, China, and manually cut into 7 × 8 cm^2^ pieces. The 10% (w/w) ham extract was prepared by placing the cut ham pieces into distilled water (1 : 10 by volume), homogenized for 2 min in a masticator (IUL Instruments, Barcelona, Spain), and then passed through 2 layers of cheese cloth. The homogenate was autoclaved to sterilize natural bacterial flora and cooled to room temperature before aseptically dispensing 100 mL into sterile glass bottles. HBA solution (45% product, brownish purple color, water soluble, density: 1.07 ± 0.01 g/mL), kindly provided by S.S. Steiner Inc. at New York, NY, was dissolved in distilled water and added to the aforementioned sterile ham extract in appropriate amounts to reach concentrations of 0, 4.44 and 10 mg/L. As previously indicated, the 100 mL of ham extract solutions was inoculated with 0.1 mL of the diluted inoculum. The inoculated glass bottles were then stored at a refrigerated incubator set at 7.2°C (Fisher Scientific, Fair Lawn, NY, stability ±0.2°C) for up to 26 days.

### 2.3. Microbiological and pH Analyses

On days 0, 3, 6, 9, 12, 16, 20, and 26 during storage, an aliquot of 5 mL solution for each treatment was 10-fold serially diluted in 0.1% peptone water and surface plated onto tryptic soy agar (Difco, Becton Dickinson), supplemented with 0.6% yeast extract (Acumedia, Lansing, MI; TSAYE) and PALCAM agar for enumeration of* L. monocytogenes* in a support medium and a selective medium, respectively. Colonies were counted manually after incubation at 30°C for 48 h with a detection limit of 0.5 log CFU/mL. Following microbial analysis, the pH of the homogenate was measured using a digital pH meter (Fisher Scientific, Fair Lawn, NY).

### 2.4. Sensory Evaluation

Sensory analysis was performed by evaluating consumer acceptability of unheated, fresh purchased RTE ham after dipping into HBA solutions. The fresh purchased ham was sliced in a Hobart 2712 12′′ semiautomatic slicer (Hobart Mfg. Co., Troy, OH) and manually cut into pieces of 7 cm × 8 cm per side with total surface area of 112 cm^2^. The ham slices were left untreated (control) or were immersed in 0 (distilled water), 0.05, 0.11, and 0.23% HBA solutions to reach the residual HBA concentrations of 0, 2.0, 4.44, and 10 mg/kg on the product surface. The dipping treatment was applied by immersing 20 pieces of ham in 250 mL of prepared HBA solution for 2 min, followed by draining for 1 min, vacuum packaging (A300/16, Multivac Inc., Germany), and overnight storage at 4°C. To verify absorption of HBA on the surface of ham (mg/kg), preliminary experiments were conducted to determine the weight gained by each ham piece after 2 min of dipping into distilled water followed by draining for 1 min as described in the previous study [15].

The sensory evaluation tests were approved by the Jiang Nan University Institutional Review Board (IRB) and were conducted in a state-of-the-art sensory laboratory. A random coded three-digit number was assigned to each sample to identify treatment groups. An untrained panel of 30 consumers was recruited from the School of Food Science and Technology at Jiang Nan University (Wuxi, Jiangsu, China) to evaluate hams for appearance, color, odor, flavor, texture, and overall acceptability. Room temperature water in plastic cups and fresh unsalted crackers were provided to each panel member to clean their palates well between samples. A 9-point hedonic scale, where 1 indicates dislike extremely and 9 indicates like extremely, was used to evaluate the appearance, odor, flavor, and overall acceptability of hams. The color (1 indicating extremely pale and 9 indicating extremely dark) and texture (1 indicating extremely soft and 9 indicating extremely firm) of hams were also evaluated.

### 2.5. Statistical Analysis and Data Modeling

The experiment was performed twice, and for each replication three individual samples were analyzed at each sampling time (*n* = 6). The pH and microbiological data (converted to log CFU/mL) were analyzed using the Mixed Model Procedure of SAS with independent variables including type of stress, treatment, time, and interactions between two and three independent factors. Results of sensory evaluation were analyzed using One-Way ANOVA of SAS. Means and standard deviations were calculated, and the differences among subgroup means were separated using a LSD adjustment for multiple comparison at the significance level of *α* = 0.05. USDA-Integrated-Predictive-Modeling-Program (IPMP) [17] and DMFIT software (Institute of Food Research, Reading, UK) were used to estimate parameters of the pathogen cells' survival/growth curve during storage. For each model, the six repeats of experimental data were used to estimate, through the root mean square error (RMSE) and Akaike Information Criterion (AIC), how well the model predicted the data.

## 3. Results and Discussion

### 3.1. Survival/Growth of Unstressed or Stress-Adapted* L. monocytogenes* in Ham Extract after Exposure to HBA

**Figure 1:**
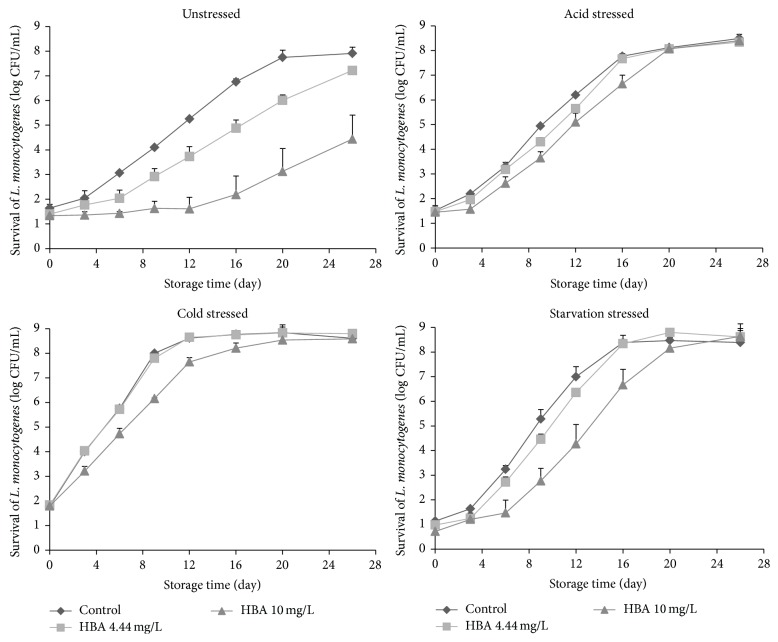
Means (SD, log CFU/mL) of unstressed and acid-, cold-, and starvation-stressed* L. monocytogenes* (tryptic soy agar with 0.6% yeast extract) populations in ham extract containing 0.0 (control), 4.44, and 10.0 mg/L hops beta acids (HBA) during storage at 7.2°C for 26 days.

**Figure 2:**
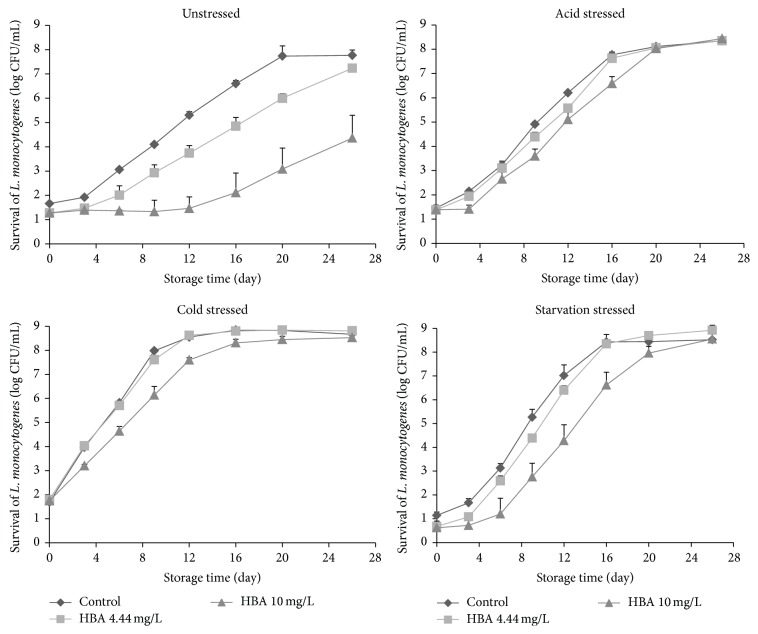
Means (SD, log CFU/mL) of unstressed and acid-, cold-, and starvation-stressed* L. monocytogenes* (PALCAM agar) populations in ham extract containing 0.0 (control), 4.44, and 10.0 mg/L hops beta acids (HBA) during storage at 7.2°C for 26 days.

Recently, the U.S. National Advisory Committee on Microbial Criteria for Foods suggested that the evaluation of antimicrobial agents inhibiting* L. monocytogenes* growth on RTE meat products should include a temperature of 45°F (7.2°C), which reflects the real RTE meat processing environment [9]. In this study, the growth behavior of unstressed or acid-, cold-, or starvation-stress-adapted* L. monocytogenes* in ham extract containing 4.44 or 10.0 mg/L of HBA was evaluated during storage at 7.2 ± 0.2°C. Throughout the 26 days of storage, the* L. monocytogenes* populations for all treatments on TSAYE (Figure 1) did not differ (*P* > 0.05) from those observed on PALCAM agar (Figure 2), indicating that the majority of* L. monocytogenes* can recover and grow on selective agar and HBA did not cause injury of the pathogen cells [18]. Therefore, the* L. monocytogenes* populations derived from the PALCAM agar were used to describe the growth dynamics of all treatments.

The initial unstressed and acid-, cold-, and starvation-stressed* L. monocytogenes* populations were 1.7, 1.5, 1.7, and 1.3 log CFU/mL, respectively. As expected, the unstressed and 3 types of stress-adapted pathogen cells grew rapidly and reached 7.8 to 8.7 log CFU/mL by the end of storage (Figure 2). At day 26, the final population of acid-, cold- and starvation-stressed cells averaged 0.8 log CFU/mL greater (*P* < 0.05) than that of the unstressed cells (Figure 2).

After exposure to HBA, the survival/growth behavior of unstressed and 3 types of stress-adapted cells was different (*P* < 0.05). For unstressed pathogenic cells, no immediate reduction was observed in 4.44 and 10 mg/L HBA solutions (Figure 2), which agrees with previous studies by Shen and Sofos [14], who reported that the initial amounts of* L. monocytogenes* in all treatments with or without HBA ranged from 2.6 to 2.8 log CFU/mL. During storage at 7.2°C, the unstressed* L. monocytogenes* growth was inhibited by HBA and this inhibition increased with increasing concentrations of HBA, which is in agreement with previous studies by Shen and Sofos [14] and Shen et al. [15]. The inhibition continued for up to 6 days and 12 days for 4.44 and 10 mg/L of HBA, resulting in 0.6 (*P* > 0.05, 4.44 mg/L of HBA) and 3.3 log CFU/mL (*P* < 0.05, 10.0 mg/L of HBA) lower pathogen populations compared to the control by the end of 26-day storage (Figure 2). The mode-of-action of HBA for the inhibition of* L. monocytogenes* growth is attributed mainly to the leakage of the cytoplasmic contents, the release of protons with a proton motive force depletion, the drop of intracellular pH, and the inhibition of the active transport of sugar and amino acids [19].

In general, after exposure to 4.44 or 10.0 mg/L of HBA, the acid-, cold- and starvation-stress-adapted* L. monocytogenes* cells showed fast growth and high final pathogen populations, ranging from 8.3 to 8.4, 8.5 to 8.8, and 8.6 to 8.9, respectively (Figure 2). Among the three types of stress-adapted cells, the growth curves of 4.44 mg/L HBA-treated samples were very similar to the control (Figure 2), which shows an apparent loss of inhibitory activity of 4.44 mg/L HBA to various stressed cells. During storage, a lower (*P* < 0.05) pathogen growth was noticed in 10.0 mg/L HBA-treated samples than in those from the control, particularly for cold- and starvation-stressed cells. However, this inhibition was much lower (*P* < 0.05) than that observed in unstressed cells. Therefore, a cross protection effect obviously developed among acid-, cold- or starvation-stress-adapted cells when exposed to HBA.

The strategies used by microorganisms to resist acid stress include pH homeostasis, changes in membrane structure by alteration of protein permeability, internal buffering ability, and the pH stability of essential proteins [20]. To survive in a cold-stress environment, bacterial cells usually modify the cell membrane to maintain membrane fluidity and macromolecular structural integrity in proteins and ribosomes [21, 22] and synthesize cold shock proteins [23]. Starvation stress causes an increase in cellular resistance capability by means of the use of alternative growth substrates or energy to stabilize ribosomes against degradation [24], change morphological transformation into spherical conformations [25], and enhance metabolic potential of microorganisms [26]. It is reported that starvation proteins are encoded by two groups of genes, including* cst *genes controlled by carbon starvation and* pex *genes controlled by carbon, nitrogen, or phosphorus starvation [26].

### 3.2. Data Modeling

In a preliminary study, four reduced and four full growth models in the USDA-IPMP software were used to evaluate the fitness of the model to predict the growth kinetics of cells in different treatments (i.e., low value of RMSE and AIC). As shown in Table 1, the Baranyi Full Growth Model fitted well growth data for all treatments based on their low RMSE (from 0.152 to 0.524) and low AIC scores (from −12.545 to −99.284). Therefore, the DMFIT software, based on Baranyi Full Growth Model, was used to compare the lag phase periods and growth rates in all treatments (Table 2).

For unstressed* L. monocytogenes* cells, reduced growth rates of 0.29 and 0.24 log CFU/mL/day were obtained in 4.44 and 10.0 mg/L HBA-treated ham extract (Table 2), respectively, which were lower (*P* < 0.05) than in ham extracts without HBA (control, growth rates of 0.39 log CFU/mL/day, Table 2). Corresponding to the microbiological data (Figures 1 and 2), HBA treatments extended the lag phase time from 2.49 days in controls to 3.49 (*P* > 0.05) and to 12.98 days (*P* < 0.05) in 4.44 and 10.0 mg/L HBA treatments, respectively (Table 2). These findings verified that HBA had promising bacteriostatic effects for unstressed* L. monocytogenes* cells in ham extract.

In the absence of HBA, the three types of stress-adapted cells behaved differently (*P* < 0.05) during storage with the calculated growth rates increasing as 0.39 (unstressed) < 0.50 (acid) < 0.68 (starvation) ≤ 0.71 (cold) log CFU/mL/day and lag phase periods decreasing as 3.26 (starvation) ≥ 2.49 (unstressed) ≥ 2.33 (acid) > undetected (cold) (Table 2). The longer lag phase time shown in starvation-stressed cells can be explained by the “shift-up” effect [27]. After transferring* L. monocytogenes* cells from 0.85% salt solution (starvation stress) to the ham extract, the cells needed extra time to construct new ribosome to enhance their ability for protein synthesis, resulting in a longer lag phase time [27]. Overall, after exposure to HBA, compared to the unstressed* L. monocytogenes* cells, acid-, cold- or starvation-stress-adapted pathogen cells showed higher (*P* < 0.05) growth rates, indicating cross protection effects. Specifically, among the 4.44 and 10.0 mg/L HBA treatments, the growth rates increased as 0.24 to 0.29 (unstressed) < 0.44 to 0.48 (acid) < 0.50 to 0.65 (cold) ≤ 0.57 to 0.66 (starvation) log CFU/mL/day (Table 2). The lag phase periods were similar (*P* > 0.05) in most HBA treatments regardless of various types of stress (Table 2). There was an inhibitory function of HBA on stress-adapted cells, especially for 10.0 mg/L HBA. For example, the growth rates of 10.0 mg/L HBA-treated cold- and starvation-stressed cells (0.50 and 0.57 log CFU/mL/day) were significantly lower than those from the control and 4.0 mg/L HBA treatment.

### 3.3. pH Variation of Ham Extract

**Figure 3:**
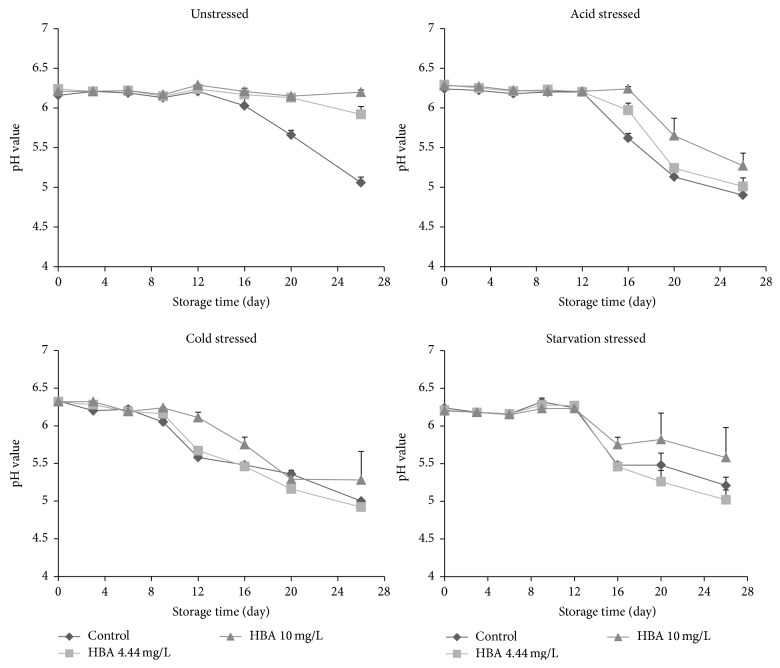
Mean (SD) pH values of ham extract containing 0.0 (control), 4.44, and 10.0 mg/L hops beta acids (HBA) during storage at 7.2°C for 26 days.

The average initial pH of untreated ham extract solution on day 0 was 6.16, while after adding HBA, the pH slightly increased to 6.21 to 6.24 (Figure 3), which is in agreement with the previous study [14]. As expected, the pH of the ham extract solution during 26 days of storage decreased significantly in samples in which significant growth (>6-7 log CFU/mL) of the unstressed or stress-adapted* L. monocytogenes* occurred in ham extract solution. The pH decrease was attributed to the microbial metabolism of carbohydrates of ham extract, generating acid into ham extract solutions in which significant growth of the pathogen population was observed [18]. For unstressed pathogen cells, the pH of HBA-treated samples did not change significantly (*P* ≥ 0.05) until 20 to 26 days of storage (Figure 3), suggesting that the unstressed* L. monocytogenes* did not grow rapidly in the presence of HBA. However, a dramatic decrease of pH value occurred at days 16 to 20, 9 to 16, and 16 for acid-, cold-, and starvation-stress-adapted-cells, respectively, regardless of control or HBA treatments (Figure 3), indicating rapid pathogen growth due to their resistance to HBA.

### 3.4. Sensory Evaluation

In an early study reported by Shen et al. [15], dipping frankfurters into 0.06 to 0.10% HBA solutions inhibited* L. monocytogenes* for 30 to 50 days of vacuum sealed storage at 4 or 10°C; however, it raised the concern that applying HBA on RTE meats might cause adverse sensory effects. Therefore, in this study, sensory evaluation was performed on unheated RTE hams dipped into 0.05 to 0.23% HBA followed by 24-h vacuum package storage at 4°C. The 30 participating panelists were primarily university food science graduate students (90%) with the age from 21 to 30 years (82%), 55% of whom were male. More than half of the participants (65%) indicated that they liked to eat RTE deli meats and frankfurters, and 50% and 45% of them ate RTE meats one to three times per month and one to five times per year, respectively. The slightly greater standard deviations that ranged from 0.56 to 1.39 (Table 3) were expected in this study, because an untrained consumer style panel was used to perform the sensory evaluation analysis.

The average hedonic scores of untreated ham were 5.68–6.28 across items of appearance, color, odor, flavor, texture, and overall acceptability (Table 3). After dipping into distilled water or 0.05 or 0.11% HBA, slightly (*P* > 0.05) lower scores were obtained among 30 panel members compared to those obtained from the untreated samples. In general, immersing ham in 0.05 and 0.11% HBA did not cause any negative effects on the tested sensory attributes, ranking between “neither like nor dislike” and “like slightly” (Table 3). Color scores were only slightly lower (*P* > 0.05), from 5.11 to 5.19, in 0.05 and 0.11% HBA-dipped hams; thus HBA did not bring severe brownish color to the ham surfaces. However, the lowest mean hedonic scores of all sensory attributes were seen in the 0.23% HBA-treated samples (Table 3). HBA is derived from hops flowers used for beer brewing process and therefore has the potential to impart a “beer like” bitter taste. Adding too much HBA into postlethality dipping solutions may decrease sensory acceptability of treated RTE meat products. In a previous study, Geornaras et al. [28] found that commercial ham and frankfurters treated with organic acids such as lactic and acetic acids resulted in significant lower hedonic scores compared to untreated controls. In this study, 0.05 and 0.11% HBA-treated samples had only slightly lower sensory scores in all attributes. Therefore, it is suggested that HBA did not cause extra adverse sensory effects as compared to the other widely used organic acid base antimicrobials. Thus, 0.11% HBA, which generated a residual concentration of 4.44 mg/kg HBA on the RTE meat surfaces (recommended by USDA-FSIS), should be confidently applied during postlethality RTE meat processing.

## 4. Conclusion

Results of the present study indicated that HBA exhibited promising inhibitory effects for unstressed* L. monocytogenes* in ham extract stored at 7.2°C. The sensory evaluation results confirmed that applying 4.4 mg/kg of HBA on RTE hams (approved by USDA-FSIS and US-FDA) will not cause adverse sensory effects. However, acid-, cold-, and starvation-stress-adapted* L. monocytogenes* cells showed resistance and cross protection to HBA. For industrial RTE meat processers, challenge studies should examine whether meat decontamination with organic acids or cold storage may provide cross protection of* L. monocytogenes* to subsequent exposure to antimicrobials, such as HBA, during postlethality processing of RTE meats. Future studies are needed to evaluate the antilisterial activities of HBA on more RTE products such as uncured or cured ham, turkey breast, and roast beef.

## Figures and Tables

**Table 1:** Comparison of RMSE and AIC for the proposed models fitting the growth of unstressed or acid-, cold-, and starvation-stress-adapted *Listeria monocytogenes* in the presence of hops beta acids (HBA) in ham extract.

Stress types	HBA (mg/L)		Reduced growth model				Full growth model			
			No lag phase	Reduced Huang	Reduced Barany	Buchanan two-phase linear	Huang	Baranyi	Modified Gompertz	Buchanan three-phase linear
Unstressed	0	RMSE	0.346	0.593	0.580	0.593	0.234	0.238	0.265	0.211
		AIC	−44.214	−18.300	−19.320	−18.300	−61.009	−60.207	−55.032	−66.168
	4.44	RMSE	—	0.299	0.297	0.300	0.290	0.291	0.290	—
		AIC	—	−51.095	−48.294	−51.022	−50.866	−50.623	−50.713	—
	10.0	RMSE	—	0.629	0.635	0.629	—	0.643	—	—
		AIC	—	−15.486	−15.008	−15.474	—	−12.545	—	—

Acid	0	RMSE	0.291	—	0.738	0.804	0.178	0.152	0.184	1.161
		AIC	−52.482	—	−7.813	−3.679	−74.253	−81.857	−72.522	15.801
	4.44	RMSE	0.321	—	0.679	0.708	0.209	0.176	0.245	0.169
		AIC	−47.684	—	−11.814	−9.806	−66.629	−74.901	−58.848	−76.699
	10.0	RMSE	0.469	0.581	0.603	0.581	0.302	0.313	0.330	—
		AIC	−29.520	−19.302	−17.465	−19.306	−48.834	−47.104	−44.580	—

Cold	0	RMSE	0.158	—	0.918	1.489	—	0.162	0.249	0.691
		AIC	−81.759	—	2.708	25.898	—	−78.793	−58.080	−9.139
	4.44	RMSE	0.108	—	0.848	1.379	—	0.106	0.218	0.736
		AIC	−100.221	—	−1.093	22.216	—	−99.284	−64.410	−6.110
	10.0	RMSE	0.209	—	0.777	1.053	0.208	0.206	0.258	0.892
		AIC	−68.320	—	−5.309	9.263	−66.632	−67.189	−56.481	3.147

Starvation	0	RMSE	0.544	—	1.103	1.174	0.354	0.343	0.365	0.357
		AIC	−22.455	—	11.520	14.489	−41.242	−42.801	−39.817	−40.759
	4.44	RMSE	0.464	—	1.012	1.043	0.216	0.189	0.251	0.263
		AIC	−30.056	—	7.391	8.824	−64.852	−71.459	−57.782	−55.544
	10.0	RMSE	0.792	0.815	0.843	0.815	0.523	0.524	0.529	0.544
		AIC	−4.420	−3.016	−1.396	−3.004	−22.488	−22.429	−21.937	−20.591

*Note.* RMSE: root mean sum of squared errors; AIC: Akaike Information Criterion (the smaller the better); “—”: model is unlikely for the data.

**Table 2:** Means (S.E.) of lag phase duration (LPD) and growth rate (GR) of unstressed and acid-, cold-, and starvation-stress-adapted *L. monocytogenes* during exposure to hops beta acids (HBA) in ham extract calculated using the Baranyi Full Growth Model in DMFIT software.

Type of stress	HBA concentration	LPD (days)	GR (log⁡CFU/ml/day)	*R* ^2^
Unstressed	0.0 mg/L (control)	2.49^a^ (0.79)	0.39^a^ (0.03)	0.99
	4.44 mg/L	3.49^a^ (1.30)	0.29^b^ (0.03)	0.98
	10.0 mg/L	12.98^b^ (3.14)	0.24^b^ (0.07)	0.93

Acid	0.0 mg/L (control)	2.33^a^ (0.46)	0.50^c^ (0.02)	0.99
	4.44 mg/L	2.89^a^ (0.46)	0.48^c^ (0.02)	0.99
	10.0 mg/L	3.42^a^ (0.88)	0.44^c^ (0.03)	0.99

Cold	0.0 mg/L (control)	—	0.71^d^ (0.02)	0.99
	4.44 mg/L	—	0.65^d^ (0.01)	0.99
	10.0 mg/L	—	0.50^c^ (0.01)	0.99

Starvation	0.0 mg/L (control)	3.26^a^ (0.73)	0.68^d^ (0.07)	0.99
	4.44 mg/L HBA	3.30^a^ (0.40)	0.66^d^ (0.03)	0.99
	10.0 mg/L HBA	5.44^a^ (1.13)	0.57^c^ (0.07)	0.97

—: Lag phase duration is not detected.

^a–d^Means in the same column with the same superscript letter were not significantly different (*P* > 0.05).

**Table 3:** Sensory analysis of ready-to-eat (RTE) ham treated with hops beta acids (HBA) solution.

Treatment^a^	Appearance^b^	Color^c^	Odor^b^	Flavor^b^	Texture^d^	Overall acceptability^b^
Control	6.25 ± 0.66^a^	5.94 ± 1.03^a^	5.68 ± 1.02^a^	6.28 ± 1.28^a^	5.73 ± 1.37^a^	6.20 ± 1.12^a^
DW	5.99 ± 0.71^ab^	5.21 ± 0.86^a^	5.26 ± 0.89^ab^	5.66 ± 1.39^a^	5.10 ± 0.80^a^	6.14 ± 0.93^a^
0.05% HBA	5.90 ± 0.58^ab^	5.11 ± 0.78^a^	5.33 ± 0.84^ab^	5.46 ± 1.13^a^	6.01 ± 0.82^a^	5.64 ± 1.36^a^
0.11% HBA	6.03 ± 0.82^ab^	5.19 ± 0.56^a^	5.14 ± 0.67^ab^	5.48 ± 1.15^a^	6.06 ± 0.80^a^	5.86 ± 0.86^a^
0.23% HBA	5.24 ± 0.77^b^	4.85 ± 0.85^a^	4.71 ± 0.57^b^	4.58 ± 0.92^a^	5.33 ± 0.80^a^	4.51 ± 1.01^a^

^a^Means with the same letter were not significantly different (*P* > 0.05).

^b^1 = dislike extremely; 9 = like extremely.

^c^1 = extremely pale; 9 = extremely dark.

^d^1 = extremely soft; 9 = extremely firm.
